# Interspecific Conformity and Asymmetric Behavioral Convergence in *Drosophila*

**DOI:** 10.1002/ece3.73149

**Published:** 2026-03-17

**Authors:** Kaiya Hamamichi, Yuma Takahashi

**Affiliations:** ^1^ Graduate School of Science and Engineering Chiba University Chiba Japan; ^2^ Graduate School of Science Chiba University Chiba Japan

**Keywords:** activity, *Drosophila takahashii*, group‐level characteristics, influencer, mixed‐species group

## Abstract

Many animals form mixed‐species groups. Interactions among individuals within a group often induce behavioral conformity, forming group‐level characteristics. However, mixed‐species groups are often studied with a focus on specific species, and it remains unclear whether conformity emerges or group‐level characteristics form in passive aggregations, such as insects patchily distributed on food sources. Here we focused on four sympatric *Drosophila* species, i.e., *D. simulans* , *D. suzukii*, *D. lutescens* , and *D. takahashii*, and revealed interspecific conformity in activity and the formation of group‐level characteristics. Within each species' solitary group, activity levels increased in other individuals when the focal individual began walking, indicating that activity conforms among individuals in all species. Experiments were conducted with *D. lutescens* in mixed groups that combined the other three species. Convergence in activity levels due to conformity was observed across all species. Interestingly, *D. takahashii* exhibited smaller behavioral changes in mixed groups than the other species. Conversely, *D. lutescens* showed the greatest change in activity levels when mixed with this species. These results suggest that *D. takahashii* exerts disproportionate influence on the formation of group‐level characteristics. Understanding intraspecific behavioral variation and intergroup variation may require considering the impact of such influential species within the same guild.

## Graphical abstract

Many animals form mixed‐species groups, but it remains unclear whether behavioral conformity and group‐level characteristics emerge in passive aggregations such as insects on shared food sources. Studying four sympatric *Drosophila* species revealed interspecific conformity in activity, with convergence of activity levels in mixed‐species groups and especially strong influence from *D. takahashii*, which induced large changes in *D. lutescens* . These results indicate that particular species can exert disproportionate effects on group‐level characteristics, highlighting the importance of species composition in understanding behavioral variation.

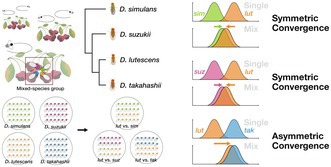

## 1 Introduction

Animals frequently form aggregations of individuals. Such aggregations may arise actively through mutual attraction among individuals, or passively when they are drawn toward favorable sites or resources that attract individuals (Bengtsson 2008). While aggregations are generally composed of a single species, mixed‐species groups also occur in nature (Goodale et al. 2017). Mixed‐species groups have traditionally been described primarily in particular taxa (Stensland et al. 2003; Mangini et al. 2023; Webster et al. 2025), but since multiple species belonging to the same guild are often attracted to the same resources, mixed‐species groups may represent a more widespread phenomenon observed across diverse taxonomic groups, and we define also such aggregations as mixed‐species groups here (Carlson et al. 2023). Indeed, although mixed‐species groups can increase the risks associated with inter‐individual competition, they also provide a variety of well‐known benefits (Beauchamp 2014). These benefits include not only dilution effects but also information exchange among individuals of different species, and the presence of other species can modify the behavioral patterns of each species (Daoudi‐Simison et al. 2023), thereby enhancing fitness. For example, social learning enhances individual foraging efficiency and helps individuals avoid predators (Avarguès‐Weber et al. 2013). Furthermore, synchronized breeding distributes predation pressure within an aggregation, leading to increased reproductive success in individuals (Robinson et al. 2013). Consequently, the fitness of individuals that form mixed‐species groups is largely influenced by the behavioral traits of individuals comprising the aggregation.

The changes in individual behavior arising from inter‐individual interactions occur throughout an aggregation, leading to the formation of group‐level characteristics (Killen et al. 2017; Voorhees 2023). Group‐level characteristics that emerge through changes in individual behavior can, in turn, feedback to alter individual behavior and fitness, thereby potentially shaping the ecological performance of mixed‐species groups (Carlson et al. 2023; Goodale et al. 2020). Therefore, understanding the mechanisms underlying the formation of group‐level characteristics in mixed‐species groups holds significant importance in ecology and evolutionary biology. Conformity, a behavioral adjustment in which an individual aligns its behavior with that of others and is a typical example of behavioral modification based on inter‐individual interactions (Claidière and Whiten 2012), is one of the mechanisms by which group‐level characteristics emerge through intraspecific interactions (Jolles et al. 2020). In addition, conformity is considered an ecologically important trait and an adaptive mechanism because it enables individuals to optimize social information use, maintain behavioral coordination, facilitate collective decision‐making, and enhance social cohesion within groups (Toyokawa and Gaissmaier 2022; Harrison et al. 2024; McCormick et al. 2024). Conformity levels vary among species (Greggor et al. 2017), suggesting that behavioral changes in individuals and group‐level characteristics resulting from conformity may exhibit greater diversity depending on species composition. However, the presence and consequences of interspecific conformity remain unconfirmed in passive mixed‐species groups observed in many invertebrates. To understand how behavioral phenotypes of individuals are determined, or how the group‐level characteristics of a mixed‐species group emerge, it is essential to elucidate both the composition of group members and the conformity‐mediated behavioral changes that occur within mixed‐species groups (Kaufhold and Van Leeuwen 2019).

*Drosophila* are model organisms for behavioral observation. In the wild, various *Drosophila* species are often attracted to the same fruits (Soto‐Yéber et al. 2018), where they may form mixed‐species aggregations. In some species, individuals actively associate with one another, forming cohesive groups through mutual attraction (Bartelt et al. 1985). *Drosophila* are considered relatively social insects (Billeter et al. 2025), and behavioral conformity among individuals through social interactions has been reported (Mery et al. 2009; Battesti et al. 2012; Kacsoh et al. 2015). The tendency to aggregate also varies among species (Jezovit et al. 2020). Because fruit flies are amenable to experimental manipulation under controlled laboratory conditions, they are also well suited for observing and analyzing mixed‐species groups. In the present study, we aimed to investigate the relationship between species composition and emergent group‐level characteristics in mixed‐species groups and the degree of conformity at both the species and group levels. Here we focused on four closely related *Drosophila* species ( *D. lutescens* , *D. simulans* , *D. suzukii*, and *D. takahashii*). In general, these *Drosophila* species are known to use fruit resources as feeding substrates, suggesting that the flies encounter and interact with one another on these resources in the wild, which may facilitate the use of information from heterospecific individuals. In natural habitats, these *Drosophila* species frequently occur in mixed‐species groups, providing an opportunity to study how behavioral conformity emerges under increasing community complexity. First, we quantified the level of conformity in each species, and then we examined changes in activity level in mixed‐species groups of two species.

## 2 Materials and Methods

### 2.1 Study Species and Rearing

We focused on *D. lutescens* , *D. simulans* , *D. suzukii*, and *D. takahashii*, all collected from the same population (Chiba University: 35°37′34″ N, 140°6′9″ E) and maintained as established strains. These species were selected based on their sympatric occurrence, phylogenetic proximity within the *melanogaster* species group, and compatibility with identical rearing and experimental conditions. For each species, two strains were randomly selected from those maintained in the laboratory and used for experiments conducted during 2022–2023. All species have been maintained in incubators controlled at 22°C under a 12:12 h light–dark cycle. Flies were reared in plastic vials (30 mm in diameter, 100 mm in height) on a nutritive medium prepared according to the protocol of Fitzpatrick et al. (Fitzpatrick et al. 2007) to obtain individuals for experiments. The flies used in all experiments were 1–7 days old after emergence. To obtain them, parent flies were removed from vials following a 1‐week egg‐laying period, and the offspring of both sexes were transferred to new vials immediately after emergence.

### 2.2 Experimental Design

**FIGURE 1:**
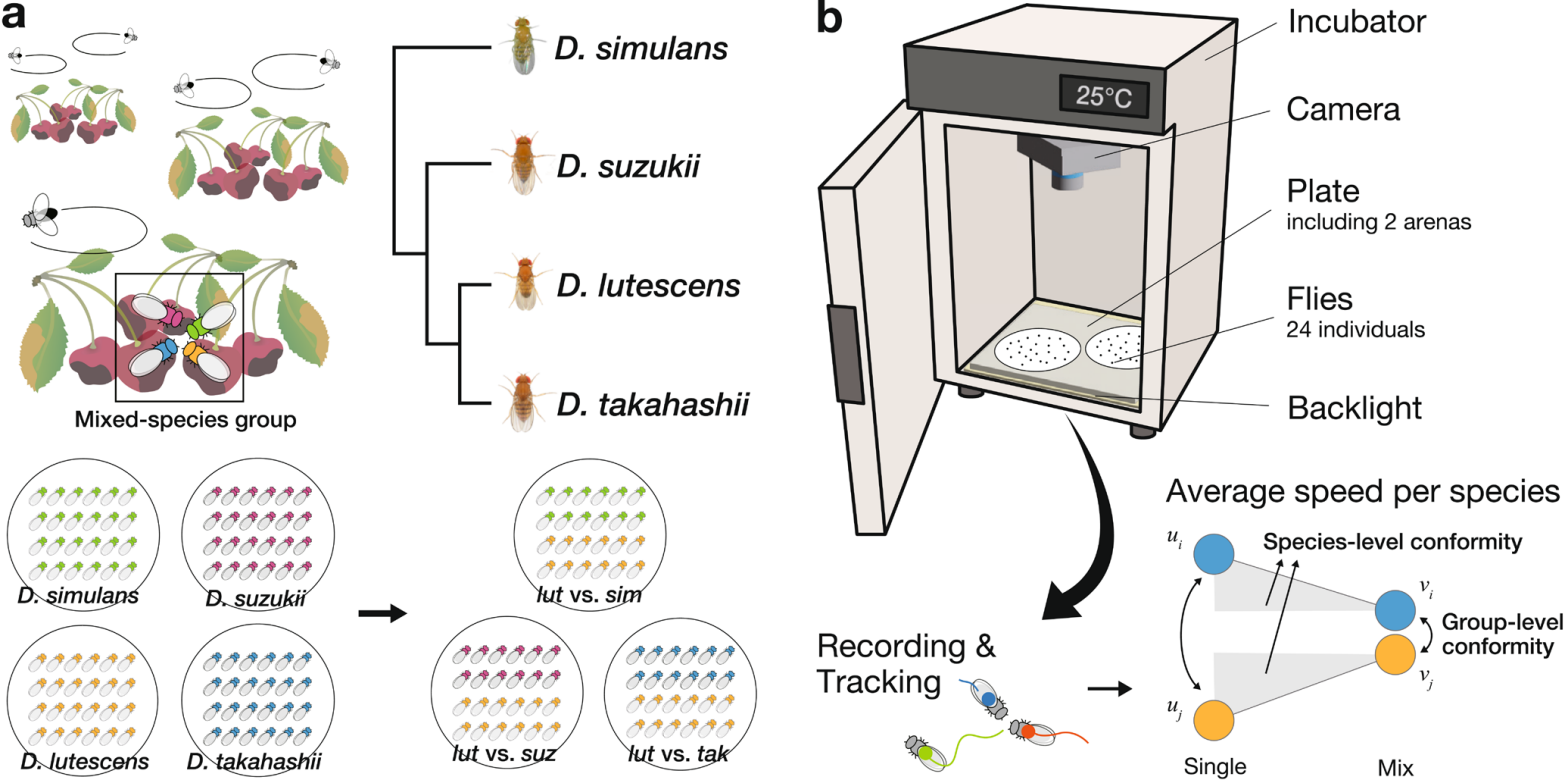
Experimental systems and methods. (a) Four species known to form mixed‐species groups by gathering at a single feeding site in the wild were used in the experiment. Each species was mixed with *D. lutescens* for the experiment. Phylogenetic relationships are shown in the molecular phylogenetic tree, with branch length not drawn to scale. Images for *D. suzukii*, *D. lutescens* , and *D. takahashii* were obtained from the website of Nicolas Gompel's laboratory; the image for *D. simulans* was obtained from KYORIN‐Fly. (b) All recordings were conducted in a controlled environment within incubators. Each recording session used one plate containing two arenas, each holding 24 flies. For the quantified locomotive speeds, the rate of change in locomotive speed from single‐species to mixed‐species groups and the change in interspecific differences in locomotive speed were defined and assessed as *species‐level* and *group‐level conformity*, respectively.

We performed all assays in a custom circular arena (140 mm diameter; 2.5 mm height), constructed to match the mortar‐type apparatus used in previous methods (Simon and Dickinson 2010). We randomly sampled female flies from the vials, anesthetized them with CO_2_, and gently transferred them into the arena. We used only females because female *Drosophila* exhibit higher sociality than males (Wice and Saltz 2021), whereas male–male behaviors (e.g., aggression, same‐sex courtship) introduce confounding interactions (Chen et al. 2002; Dukas 2010). Immediately after placement, arenas were maintained at 25°C in an incubator and behavior was recorded from above with a high‐resolution video camera. Recording continued for 60 min: the initial 30 min served as an acclimation period, and the final 30 min were analyzed. To minimize the influence of circadian rhythm on fly behavior, all recording was conducted within the 5 h preceding lights‐off. Each arena contained 24 flies and constituted a “group”: either a single‐species group (24 conspecifics) or a two‐species mixture consisting of 12 individuals per species (1:1 ratio). For single‐species trials, anesthetized flies were positioned at the arena center immediately before recording to standardize initial locations. In mixed‐species trials, 12 flies of each strain were drawn from separate vials and placed at two symmetric positions along the arena y‐axis (±y), so that the initial location represented species identity. To equalize initial geometry across single‐ and mixed‐species trials, starting positions were set nearer the arena center to reduce spatial dispersion. We first recorded single‐species groups (24 individuals) and subsequently recorded mixed‐species groups comprising 12 individuals per species. For each species, two distinct strains served as biological replicates, and we recorded 3–5 replicate groups per strain (Table S1). We focused on mixed‐species groups centered on *D. lutescens* to provide a consistent reference species (host species) across treatments (Figure 1a). In each treatment, one strain of *D. lutescens* was paired with one strain of one other species. Using two strains of *D. lutescens* and two strains of each of the three other species resulted in 12 pairwise combinations. This design allowed us to systematically compare how different heterospecific species influence locomotor behavior while controlling for strain‐specific effects through a complete 2 × 2 cross. Videos were processed using FlyTracker (Eyjolfsdottir et al. 2014), enabling automated individual tracking and species assignment based on initial positions and morphological features (Figure 1b). We extracted locomotive speed and computed visual cue metrics from the coordinate time series. We define the visual cue as the summed movement of all other flies within a forward 180° sector relative to a focal individual's orientation (Ferreira and Moita 2020; Bentzur et al. 2021; Hamamichi and Takahashi 2025; Sato and Takahashi 2025); this quantity is given by

$$\text{Visual}\;\mathrm{cue}=\sum_{i=1}^{23}{\text{speed}}_{i}\times 2\arctan\left(\frac{\text{apparent body size}}{2{\text{Distance}}_{i}}\right)$$

where ${\text{speed}}_{i}$ is the speed of the i‐th other individual, ${\text{Distance}}_{i}$ is the distance from the focal individual to individual i. Here, the apparent body size was calculated by approximating the body as an ellipse with a major axis of 3.0 mm and a minor axis of 1.5 mm, and the major axis of the ellipse was used to compute the projected width for the angular term. In a previous study, we had already obtained coordinate data from 20 strains in single‐strain groups of *D. lutescens* under the same experimental design (Hamamichi and Takahashi 2025). Therefore, in the present study, coordinate data from only the two strains used in the mixed‐strain groups were obtained and analyzed.

### 2.3 Assessment of Conformity Level

We evaluated group‐level conformity and species‐level conformity using two methods (Figure 1b), because phenotypic convergence can be achieved even if only one of the mixed species shows conformity, which necessitates a clear distinction between the observed phenotypic convergence and the contribution of each species. Group‐level conformity was assessed by analyzing locomotive speed differences between the two species in single‐ and mixed‐species groups. Species‐level conformity was calculated based on locomotive speed in single‐species groups and the proportional change in locomotive speed in mixed‐species groups. This approach allowed us to evaluate two aspects: the intrinsic conformity within each species and the overall group‐level conformity resulting from interspecies interactions.

### 2.4 Sensitivity to Visual Cue

The degree of individual‐level conformity was assessed by quantifying how much an individual's locomotive speed changed in response to changes in the visual cue. Specifically, simple linear regression was performed with locomotor speed as the dependent variable and visual cue as the independent variable. The regression coefficient was used as an indicator of sensitivity to the visual cue. Furthermore, to account for the delay before changes in visual information are reflected in behavior, the regression analysis estimated the regression coefficient by shifting the speed time series one frame per 0.5 s later relative to the visual cue (Hamamichi and Takahashi 2025). Note that visual cues were first adjusted by adding the minimum non‐zero value to zero‐valued observations, followed by log‐transformation and species‐level standardization to reduce the influence of outliers and to account for baseline differences in mean visual cue values among species.

### 2.5 Definition Of Locomotion Event

To evaluate whether activity conformity occurs in flies, we defined walking and stopping based on previous studies (see Supplementary Text and (Bentzur et al. 2021)) and assessed conformity using changes in visual cues during these states. We defined the 0.5‐s frame immediately preceding the transition from a stationary state to walking as “walk”, and the frame immediately preceding the transition from a walking state to a stationary state as “stop”. In addition, we defined the walking state outside these locomotion events as “walking”, and the stationary state as “stay”. If flies exhibit activity conformity, the visual cue is expected to change according to these locomotion events. Specifically, the visual cue is expected to increase from “stay” to “walk” and decrease from “walking” to “stop”.

### 2.6 Statistical Analyses

All statistical analyses were performed in R (version 4.3.0). The R package *R.matlab* was used to import coordinate data obtained from video tracking. Differences in response variables across locomotion event were tested using the R package *glmmTMB*. A generalized mixed linear model (GLMM) with a Tweedie distribution with a log link, including three interaction terms: strain, strain × group, and strain × group × individual, was employed. Tests were conducted using *t*‐tests with *P*‐values adjusted using the Bonferroni correction. Interspecific differences were analyzed using the R package *lme4*, employing mixed linear model (LMM) and generalized linear mixed models (GLMM) with a gamma‐distributed response variable, with strain and strain‐by‐group interaction included as random effects. Interspecific differences were evaluated using ANOVA using the R package car, with Tukey's post hoc test using the R package *emmeans* for multiple comparisons. For model fitting, restricted maximum likelihood (REML) was used for LMMs, and maximum likelihood (ML) was used for GLMMs. Note that, time‐series data of locomotive speed were summarized at the individual level. Mean locomotive speed were calculated for each individual over the analysis window, and these individual‐level values were used for all statistical tests. Time points were not treated as independent observations.

## 3 Results

**FIGURE 2:**
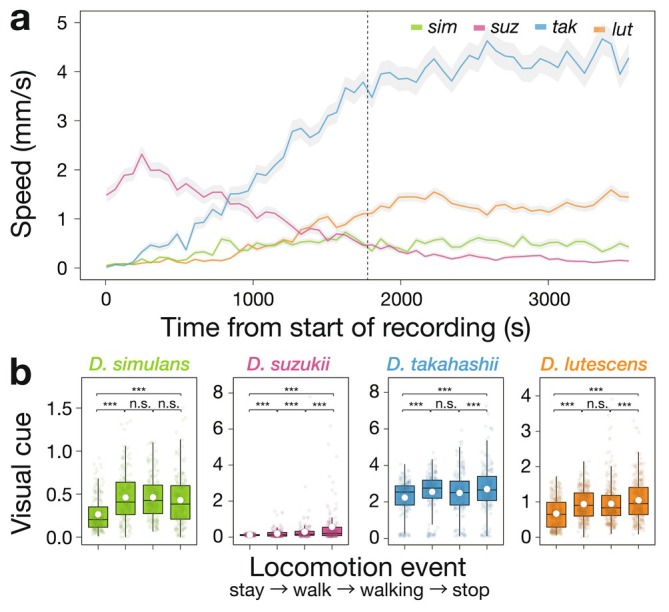
Behavioral analyses in single‐species groups. Colored points represent individual replicates, and white points indicate the mean for each boxplot. (a) The locomotive speed of each species at each time point is shown. Lines show the mean values, and the shaded gray area represents the standard error of the mean (SEM). Colors indicate each species: Green for *D. simulans* , pink for *D. suzukii*, blue for *D. takahashii, and* orange for *D. lutescens* . For clarity, data were averaged over 1 min intervals. While recovery dynamics after CO_2_ anesthesia differed among species, behavior appeared visually stable from 30 min after the start of recording onward (i.e., to the right of the vertical dashed line). Accordingly, only behavioral data collected after 30 min from the start of recording were used for the analyses. (b) Visual cues present during or immediately preceding each locomotion event are shown for each species. *P* values were adjusted using the Bonferroni method. Statistical significance: ***p < 0.001, n.s. *p* > 0.05.

The average locomotive speed significantly differed among species (*p* < 0.001), with *D. takahashii* exhibiting particularly high activity levels, approximately ten times higher than that of *D. suzukii*, the species with the lowest activity (Figure 2a). Visual cues significantly increased between “stay” and “walk” in all species ( *D. simulans* (*sim*): *p* < 0.001; *D. suzukii* (*suz*): *p* < 0.001; *D. takahashii* (*tak*): *p* < 0.001, *D. lutescens* (*lut*): *p* < 0.001), indicating conformity with nearby individuals (Figure 2b). Furthermore, while no species showed a significant change in visual cues between “walk” and “walking” (*sim*: *p* = 1.00; *suz*: *p* = 0.21; *tak*: *p* = 0.88; *lut*: *p* = 1.00), in *D. suzukii, D. takahashii*, and *D. lutescens* visual cues increased between “walking” and “stop” (*sim*: *p* = 0.34; *suz*: *p* = 0.0044; *tak*: *p* = 0.0028; *lut*: *p* = 0.0013). Between “stop” and “stay”, visual cues decreased in all species (*sim*: *p* < 0.001; *suz*: *p* < 0.001; *tak*: *p* < 0.001; *lut*: *p* < 0.001).

**FIGURE 3:**
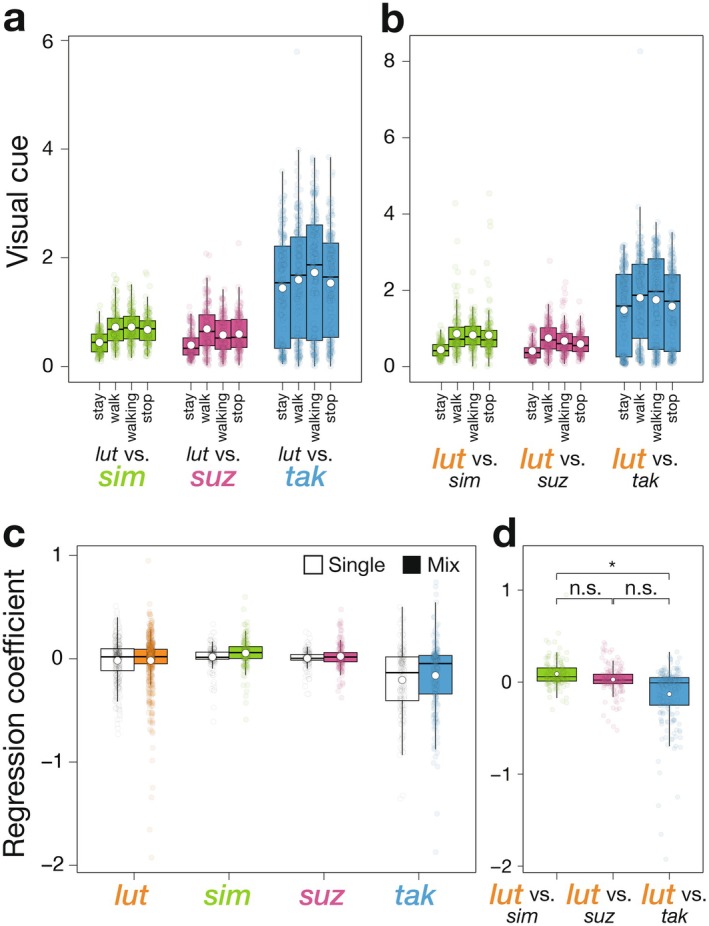
Conformity of activity across species compositions. Colored points represent individual replicates, and white points indicate the mean for each boxplot. (a) Distribution of visual cues occurring during or immediately before each locomotion event across different species compositions. *P* values were adjusted using the Bonferroni method. (b) Distribution of visual cues occurring during or immediately before each locomotion event for *D. lutescens* when paired with different heterospecific species. *p* values were adjusted using the Bonferroni method. (c) Regression coefficients derived from linear models relating visual cues (independent variable) to locomotive speed (dependent variable) in single‐species and mixed‐species groups for each species. White boxplots indicate single‐species groups, whereas colored boxplots indicate mixed‐species groups. (d) Regression coefficients for *D. lutescens* in mixed‐species groups, shown separately for each heterospecific partner. *P* values were adjusted using the Tukey method. Statistical significance: *p < 0.05, n.s. *p* > 0.05.

In mixed‐species groups, the analysis of visual cues for each locomotion event revealed a significant increase from “stay” to “walk” in *D. simulans* , *D. suzukii*, and *D. takahashii* (*p* < 0.001 for each species, Figure 3a). In *D. lutescens* , a significant increase was observed when mixed with *D. simulans* , *D. suzukii*, and *D. takahashii* (*p* < 0.001 for each species, Figure 3b). These results indicate that activity conformity occurs even in mixed‐species groups. Changes in visual cues between “walk” and “walking” depended on species composition. Significant effects were detected for *D. simulans* (*p* = 0.027) and *D. takahashii* (*p* < 0.001), but not for *D. suzukii* (*p* = 1.00) (Figure 3a). In *D. lutescens* , significant effects were found when mixed with *D. suzukii* (*p* = 0.0017) and *D. takahashii* (*p* < 0.001), whereas no effect was detected with *D. simulans* (*p* = 1.00) (Figure 3b). Similarly, changes in visual cues between “walking” and “stop” depended on species composition. Significant effects were detected for *D. suzukii* (*p* = 0.0086), but not for *D. simulans* (*p* = 0.26) and *D. takahashii* (*p* = 0.14) (Figure 3a). In *D. lutescens* , significant effects were observed with *D. suzukii* (*p* < 0.001) and *D. takahashii* (*p* < 0.001), but not with *D. simulans* (*p* = 0.80) (Figure 3b). In contrast, the change in visual cues between “stop” and “stay” was significant for *D. simulans* , *D. suzukii*, and *D. takahashii* (*p* < 0.001 for each species) (Figure 3a). A similar increase was observed in *D. lutescens* when mixed with *D. simulans* , *D. suzukii*, and *D. takahashii* (*p* < 0.001 for each species) (Figure 3b). However, the direction of change (increase or decrease) differed among species compositions. When the sensitivity to visual cue was calculated using the regression coefficient between visual cues and locomotive speed, the regression coefficient differed significantly among species (*p* = 0.0037), whereas group type (single‐ vs. mixed‐species) and species‐by‐group interactions showed no significant effects (*p* = 0.69 and *p* = 1.00, respectively; Figure 3c). Furthermore, in *D. lutescens* , analysis of heterospecific effects on conformity revealed that the regression coefficient between visual cues and locomotive speed differed significantly only between *D. simulans* and *D. takahashii* (*p* = 0.038), but not between *D. simulans* and *D. suzukii* (*p* = 0.72) nor between *D. suzukii* and *D. takahashii* (*p* = 0.13) (Figure 3d).

**FIGURE 4:**
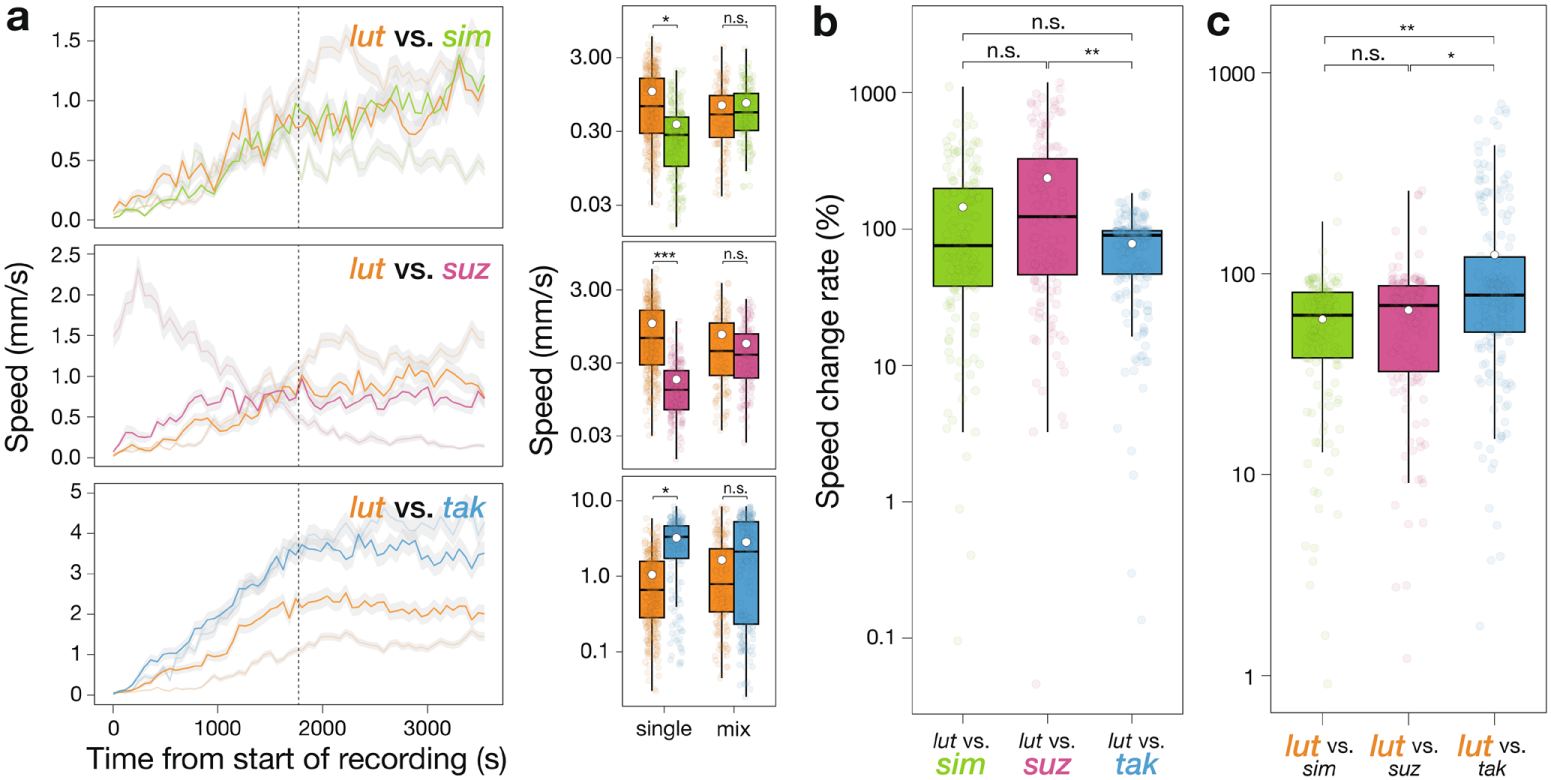
Locomotive speed in mixed‐species groups. Colored points represent individual replicates, and white points indicate the mean for each boxplot. (a) The locomotive speed of each species at each time point is shown. Lines show the mean values, and the shaded gray area represents the standard error of the mean (SEM). Colors indicate each species: Green for *D. simulans* , pink for *D. suzukii*, blue for *D. takahashii, and* orange for *D. lutescens* . The light lines indicate single‐species groups, whereas the dark lines indicate mixed‐species groups. For clarity, data were averaged over 1 min intervals. While recovery dynamics after CO_2_; anesthesia differed among species, behavior appeared visually stable from 30 min after the start of recording onward (i.e., to the right of the vertical dashed line). Accordingly, only behavioral data collected after 30 min from the start of recording were used for the analyses. Boxplots show the mean values for each species and group type (single or mixed). (b) Boxplots show the rate of change in locomotive speed from single‐species to mixed‐species groups for each species when mixed with *D. lutescens* . (c) Boxplots show the rate of change in locomotive speed from single groups to mixed groups for *D. lutescens* when mixed with each of the three species. *p* values were adjusted using the Tukey method. Statistical significance: ***p < 0.001, **p < 0.01, *p < 0.05, n.s. *p* > 0.05.

In single‐species groups, all other species differed significantly from *D. lutescens* in locomotive speed (*lut* vs. *sim: p = 0.018; lut* vs. *suz: p < 0.001; lut* vs. *tak: p = 0.011*, Figure 4a). In contrast, in mixed‐species groups, these differences disappeared in all species combinations (*lut* vs. *sim: p =* 0.88; *lut* vs. *suz: p =* 0.46; *lut* vs. *tak: p =* 0.70). These results indicate that the locomotive speeds of the two species converged when co‐occurring to a specific value for each species combination, demonstrating group‐level conformity. The change in locomotive speed of the three species when mixed with *D. lutescens* showed no significant difference between *D. simulans* and *D. suzukii* (*p =* 0.17, Figure 4b), nor between *D. simulans* and *D. takahashii* (*p =* 0.25). A significant difference was observed between *D. suzukii* and *D. takahashii* (*p =* 0.0019). These results indicate that *D. takahashii* showed the lowest species‐level conformity among the species. The change in the locomotive speed of *D. lutescens* in each mixed‐species group did not differ significantly between mixing with *D. simulans* and mixing with *D. suzukii* (*p =* 0.92, Figure 4c). Significant differences were also observed between *D. simulans* and *D. takahashii* (*p =* 0.0077), and between *D. suzukii* and *D. takahashii* (*p =* 0.026). In other words, *D. lutescens* showed the greatest species‐level conformity when mixed with *D. takahashii*. Taken together, these results indicate that *D. takahashii* exhibits a lower degree of interspecific conformity compared to other species and strongly influences the activity levels of partner species during interactions.

## 4 Discussion

The present study shows that flies adjust their activity not only to conspecifics but also to heterospecifics, resulting in more uniform activity levels among species. The conformity level varied among species, with *D. takahashii* exhibiting a smaller change in locomotive speed than other species, suggesting a lower level of conformity. Conversely, *D. lutescens* interacting with *D. takahashii* exhibited the largest change in locomotive speed, indicating that the influence on the activity of individuals of other species differs among species. In the wild, the presence of such species (*D. takahashii*) could play an influencer role, determining behavioral properties at the community level.

Four species used in the present study belong to the *melanogaster* group and are relatively closely related, yet they exhibit variation in activity levels and locomotion patterns. The change in visual cues between stationary (“stay”) and the onset of walking (“walk”) and between stopping (“stop”) and stationary (“stay”) was common across species, suggesting that all species conformed their walking behavior to others. Conversely, the change in visual cues between walking (“walking”) and stopping (“stop”) varied among species. In *D. suzukii* and *D. takahashii*, a behavior seemingly contrary to conformity‐based walking patterns was observed, with a significant increase in visual cues at stopping, suggesting that these two species exhibit higher levels of sociality, that is, a tendency to stop where many other individuals are present. In contrast, this behavioral pattern was not observed in individuals of *D. simulans* , suggesting that they exhibit lower levels of sociality compared to the other two species. On the other hand, changes in visual cues associated with these locomotion events were not necessarily consistent across species compositions. Although a significant increase in visual cues immediately before walking was observed across all species compositions in mixed‐species groups, no corresponding increase before stopping was detected for *D. lutescens* or *D. takahashii*. Specifically, in *D. lutescens* , this pattern of visual cue change was either absent or significantly reduced across all species compositions. These results suggest that, in *D. lutescens* and *D. takahashii*, walking responses to visual cues were modified by the presence of heterospecifics, which may reflect reduced social responsiveness toward non‐conspecific individuals. In addition, evaluating sensitivity to visual cues based on their relationship with locomotive speed revealed no effect of species composition (single‐ or mixed‐species groups), whereas significant differences were detected among species. However, in *D. lutescens* , sensitivity was significantly reduced when mixed with *D. takahashii* compared with *D. simulans* . This difference may be attributable to *D. takahashii* exhibiting higher locomotor speed than the other three species. In summary, conformity of locomotive speed was observed in all four species examined here and occurred even in mixed‐species groups, but although the degree of conformity, shaped by sociality and sensitivity, is largely influenced by species‐specific characteristics, it may also vary with the traits of partner species.

Indeed, interspecific differences in sociality, which directly influence contributions to the visual cues of others, as well as differences in sensitivity to visual cues, may affect the resulting changes in locomotive speed. Although there is no difference in the change in locomotive speed between *D. takahashii* and *D. simulans, D. lutescens*, when mixed with either species, showed a lower change in locomotive speed with *D. simulans* than when mixed with *D. takahashii*, suggesting that *D. simulans* may exert weaker social influence than *D. takahashii*. Furthermore, the highest change in the locomotive speed of *D. lutescens* occurred when mixed with *D. takahashii*, which had the lowest rate of change among the three species excluding *D. lutescens* , indicating that the influence on the behavior of other species may also be shaped by the degree of mutual conformity. The elimination of interspecific differences in locomotive speed across all species combinations suggests that group‐level conformity does not require mutual conformity by both species. Rather, group‐level conformity likely emerges from adjustments in species‐level conformity, shaped by the conformity and sociality of each species and by the specific partner species with which each species interacts in mixed‐species groups. Differences in conformity or sociality may shape the influence on other individuals, ultimately determining the activity level of the group. Importantly, the presence of specific species with low conformity may strongly influence the behavioral patterns of the group members.

In recent years, numerous studies have reported mixed‐species groups in diverse taxonomic groups (Stensland et al. 2003; Mangini et al. 2023; Webster et al. 2025), and their presence is increasingly recognized as a widespread phenomenon. Indeed, many studies have demonstrated conformity within mixed‐species groups (Avarguès‐Weber et al. 2013; Farine 2014; Goodale et al. 2014), showing that cooperative actions benefit individuals regardless of whether interacting partners are conspecifics or heterospecifics. Conversely, there are few verified examples of conformity among species that form non‐moving groups, such as those observed in *Drosophila*. Therefore, demonstrating that conformity can occur among such species is an important finding of the present study. Cooperative foraging among *Drosophila* species has been reported in the larval stage (Kuhar et al. 2025), and the interspecific conformity revealed in the present study may represent a precursor to such cooperative behavior. Furthermore, adult *Drosophila* are known to engage in social learning, conforming to others when selecting oviposition substrates, avoiding predators, and choosing mating partners (Mery et al. 2009; Battesti et al. 2012; Kacsoh et al. 2015). Further investigation is warranted to determine whether such fitness‐related behaviors also exhibit interspecific conformity.

The present study indirectly demonstrated differences in species' influence on conformity and the mechanisms underlying it, but there are several uncontrolled factors and limitations. We used *D. lutescens* as the host species. Although the choice of *D. lutescens* as the host species was not based on a priori differences in locomotor activity, this species exhibited intermediate activity levels across conditions. This positioning allowed us to examine how species with contrasting locomotor tendencies respond within a common social context. Furthermore, the effect of age could not be eliminated. *Drosophila* flies have been reported to exhibit age‐dependent behavioral variations (Simon et al. 2006; Iliadi and Boulianne 2010; Overman et al. 2022). Because individuals were grouped by age, this influence could not be eliminated and may have influenced changes in locomotive speed associated with conformity. Nevertheless, the occurrence of conformity between species and the differences in social influence among closely related species represent an important discovery, suggesting the existence of a novel mechanism whereby other species shape group‐level characteristics. In addition, another unconsidered possibility is that the observed conformity was not due to matching the locomotor behavior of other individuals, but rather a simple avoidance response to individuals of other species. Avoidance responses are particularly pronounced when individuals coexist with faster or larger species. However, this avoidance‐based explanation alone cannot fully account for our observations, because not only slower species exhibited increased locomotive speed, but also faster species exhibited reduced locomotive speed in mixed‐species groups. At a minimum, the phenomenon whereby faster species reduce their locomotive speed suggests that behavioral adjustments beyond simple avoidance reactions toward individuals of other species are occurring within these species.

Although the four species used here belong to the same *melanogaster* species group, differences in locomotive speed were observed, with *D. takahashii* exhibiting a particularly high activity level. These species are nearly identical in body size and were sampled sympatrically using the same bait traps, suggesting that these species share similar ecological contexts, including predator species and resources. On the other hand, although there are differences in food resources specific to *D. suzukii* and differences in cold tolerance between *D. lutescens* and *D. takahashii* (Fukatami 1984; Mitsui et al. 2010), but neither study provides logical support for interspecific differences in activity. Therefore, these behavioral differences are more likely to represent byproducts rather than the result of adaptations such as niche partitioning. On the other hand, regarding activity conformity, previous studies have argued that conformity, defined as walking in response to another individual's freezing followed by the resumption of movement, facilitates escape from predators (Sato and Takahashi 2025). Thus, a common interspecific predator avoidance strategy involving walking and stopping in response to others may have shaped convergent activity levels. However, *D. takahashii* exhibited a lower degree of conformity than the other species. This result suggests that *D. takahashii* may employ a strategy of fleeing upon detecting a predator, rather than stopping in response to others. Note that, since wild flies escape predators by flight, the link between walking and predator avoidance strategies requires careful consideration. Additionally, the habitat of *D. takahashii* is ecologically distinct compared to that of the other species. *D. takahashii* is natively subtropical (Novković and Kimura 2015), and the population used here may be opportunistic or recently established. Furthermore, *D. takahashii* is closely related to *D. lutescens* , and interspecific hybridization between them is known (Fukatami 1984), suggesting that *D. takahashii* and *D. lutescens* are potentially strong competitors. In other words, the *D. takahashii* strains used here may possess unique ecological characteristics distinct from the other three species. However, while the high activity level and low conformity of *D. takahashii* may be related to these ecological factors, further research delving into the ecology of *D. takahashii* in the wild is necessary to establish a direct link based on the results of the present study.

The present study examined how activity changes at the group level arise from interspecific conformity, demonstrating that particular species can exert a substantial influence on group‐level dynamics. Convergent changes in behavior arising from inter‐individual interactions may mask underlying genetic variation, suggesting that intraspecific behavioral variation within regions or populations may be determined more by the composition of sympatric species or by the presence or absence of particular species. Furthermore, changes in individual behavior are a crucial factor affecting both predation risk and foraging efficiency (either increased or decreased) (Sih et al. 2004). Consequently, the presence of specific species within a guild may also alter relationships between guilds. This perspective is novel within community ecology, highlighting the need within this field to explicitly consider the effects of particular species on other members of other species within the guild and on other species outside the guild.

## Author Contributions

**Kaiya Hamamichi:** conceptualization (lead), data curation (lead), formal analysis (lead), funding acquisition (supporting), investigation (lead), methodology (lead), validation (lead), visualization (lead), writing – original draft (lead), writing – review and editing (lead). **Yuma Takahashi:** conceptualization (supporting), funding acquisition (lead), project administration (lead), writing – original draft (supporting), writing – review and editing (supporting).

## Funding

This work was supported by the Sasakawa Research Grant from the Japan Science Society (2023‐5051 to K.H.).

## Ethics Statement

The authors have nothing to report.

## Conflicts of Interest

The authors declare no conflicts of interest.

## Supporting information


**Appendix S1:** Supporting Information.

## Data Availability

All data used in the present study will be made publicly available on Figshare upon publication.doi: 10.6084/m9.figshare.30758939For reviewers: https://figshare.com/s/6eed882432b4b6cb09e4.
