# Supplementary material for: Interspecific Conformity and Asymmetric Behavioral Convergence in Drosophila
Source: Ecol Evol. 2026 Mar 17;16(3):e73149. doi: 10.1002/ece3.73149 (PMC13093764; doi:10.1002/ece3.73149)
Supplement: Supplementary file 1 — Appendix S1: Supporting Information. [file ECE3-16-e73149-s001.pdf]

Supporting Information

Kaiya Hamamichi[1] and Yuma Takahashi[2]

1. Graduate School of Science and Engineering, Chiba University, Chiba, Japan

2. Graduate School of Science, Chiba University, Chiba, Japan

Corresponding author: Yuma Takahashi (E-mail: takahashi.yum@gmail.com)

Walk criteria. To prevent tracking coordinate drift caused by video quality from being misidentified as walking, locomotion was classified as walk only when the speed exceeded 0.5 mm/s. After this thresholding, walking behavior was binarized (1: walking, 0: stopping), where walking events lasting less than one second were assigned a value of 0. Finally, if walking resumed within 4 seconds after its termination, the events were regarded as a single continuous walking sequence (1).

Table S1. All sample information used in the analysis.

date	time	ID	arena	line1	line2	age	species
9-Aug-22	15:49:00	20220809_DJI_03	a01	NA	L077	4_7	lut
9-Aug-22	15:49:00	20220809_DJI_03	a02	NA	L077	4_7	lut
10-Aug-22	15:40:00	20220810_DJI_03	a01	NA	L575	1_5	tak
10-Aug-22	15:40:00	20220810_DJI_03	a02	NA	L575	1_5	tak
10-Aug-22	16:41:00	20220810_DJI_04	a01	NA	L421	1_5	tak
10-Aug-22	16:41:00	20220810_DJI_04	a02	NA	L421	1_5	tak
12-Aug-22	16:26:00	20220812_04	a01	NA	L077	1_3	lut
12-Aug-22	16:26:00	20220812_04	a02	NA	L077	1_3	lut
14-Aug-22	14:49:00	20220814_03	a01	NA	L077	3_5	lut
14-Aug-22	14:49:00	20220814_03	a02	NA	L077	3_5	lut
15-Aug-22	13:18:00	20220815_DJI_01	a01	NA	L575	3_5	tak
15-Aug-22	13:18:00	20220815_DJI_01	a02	NA	L575	3_5	tak
17-Aug-22	14:51:00	20220817_03	a01	NA	L137	2_5	lut

17-Aug-22	14:51:00	20220817_03	a02	NA	L137	2_5	lut
23-Aug-22	13:58:00	20220823_02	a02	NA	L137	1_4	lut
23-Aug-22	15:07:00	20220823_03	a02	NA	L137	1_4	lut
30-Aug-22	15:43:00	20220830_DJI2_03	a02	NA	L137	1_4	lut
11-Nov-22	14:50:00	20221111_DJI_01	a01	L077	L421	3_6	lut_tak
11-Nov-22	14:50:00	20221111_DJI_01	a02	NA	L421	3_6	tak
11-Nov-22	16:52:00	20221111_DJI_02	a01	L077	L421	3_6	lut_tak
11-Nov-22	16:52:00	20221111_DJI_02	a02	L077	L421	3_6	lut_tak
11-Nov-22	17:07:00	20221111_DJI2_02	a01	L077	L421	3_6	lut_tak
11-Nov-22	17:07:00	20221111_DJI2_02	a02	L077	L421	3_6	lut_tak
10-Feb-23	13:00:00	20230210_DJI_01	a02	NA	L575	3_6	tak
25-Aug-23	13:49:00	20230825_DJI_01	a01	NA	L004	1_3	suz
25-Aug-23	13:49:00	20230825_DJI_01	a02	L001	L077	1_3	lut_sim
25-Aug-23	14:03:00	20230825_DJI2_01	a01	NA	L001	1_3	sim
25-Aug-23	14:03:00	20230825_DJI2_01	a02	L001	L077	1_3	lut_sim
29-Aug-23	13:49:00	20230829_DJI2_01	a01	NA	L001	1_4	sim
29-Aug-23	13:49:00	20230829_DJI2_01	a02	L001	L077	1_4	lut_sim
29-Aug-23	15:05:00	20230829_DJI_02	a01	NA	L002	1_4	sim
29-Aug-23	15:05:00	20230829_DJI_02	a02	NA	L003	1_4	suz
29-Aug-23	14:53:00	20230829_DJI2_02	a01	NA	L001	1_4	sim
29-Aug-23	14:53:00	20230829_DJI2_02	a02	NA	L004	1_4	suz
1-Sep-23	14:39:00	20230901_DJI_01	a01	NA	L003	4_7	suz
1-Sep-23	14:39:00	20230901_DJI_01	a02	L003	L077	1_3	lut_suz

1-Sep-23	15:06:00	20230901_DJI2_01	a01	NA	L002	1_3	sim
1-Sep-23	15:06:00	20230901_DJI2_01	a02	L002	L077	1_3	lut_sim
1-Sep-23	16:12:00	20230901_DJI_02	a01	NA	L003	1_3	suz
1-Sep-23	16:12:00	20230901_DJI_02	a02	NA	L001	1_3	sim
1-Sep-23	16:36:00	20230901_DJI2_02	a01	NA	L001	1_3	sim
1-Sep-23	16:36:00	20230901_DJI2_02	a02	L077	L575	1_3	lut_tak
5-Sep-23	14:53:00	20230905_DJI_01	a01	NA	L004	1_4	suz
5-Sep-23	14:53:00	20230905_DJI_01	a02	L004	L077	1_4	lut_suz
5-Sep-23	15:10:00	20230905_DJI2_01	a01	L077	L575	1_4	lut_tak
5-Sep-23	15:10:00	20230905_DJI2_01	a02	L077	L575	1_4	lut_tak
7-Sep-23	14:28:00	20230907_DJI_01	a01	L004	L077	3_6	lut_suz
7-Sep-23	14:28:00	20230907_DJI_01	a02	L003	L077	3_6	lut_suz
7-Sep-23	14:46:00	20230907_DJI2_01	a01	L002	L077	3_6	lut_sim
7-Sep-23	14:46:00	20230907_DJI2_01	a02	L003	L077	3_6	lut_suz
7-Sep-23	16:02:00	20230907_DJI_02	a01	NA	L002	3_6	sim
7-Sep-23	16:02:00	20230907_DJI_02	a02	L002	L077	3_6	lut_sim
6-Oct-23	14:02:00	20231006_DJI2_01	a01	L137	L421	1_3	lut_tak
6-Oct-23	14:02:00	20231006_DJI2_01	a02	L137	L421	1_3	lut_tak
6-Oct-23	15:01:00	20231006_DJI_02	a01	L077	L575	1_3	lut_tak
6-Oct-23	15:01:00	20231006_DJI_02	a02	L077	L575	1_3	lut_tak
11-Oct-23	15:04:00	20231011_DJI_01	a02	L004	L137	1_5	lut_suz
11-Oct-23	15:26:00	20231011_DJI2_01	a01	L003	L137	1_5	lut_suz
11-Oct-23	16:10:00	20231011_DJI_02	a01	L002	L137	1_5	lut_sim

11-Oct-23	16:28:00	20231011_DJI2_02	a01	L001	L137	1_5	lut_sim
13-Oct-23	13:50:00	20231013_DJI_01	a01	L077	L004	3_7	lut_suz
13-Oct-23	14:03:00	20231013_DJI2_01	a01	L137	L004	3_7	lut_suz
13-Oct-23	14:03:00	20231013_DJI2_01	a02	L137	L575	3_7	lut_tak
13-Oct-23	15:37:00	20231013_DJI2_02	a01	L137	L003	3_7	lut_suz
13-Oct-23	15:37:00	20231013_DJI2_02	a02	L137	L003	3_7	lut_suz
13-Oct-23	17:18:00	20231013_DJI_03	a01	L137	L575	3_7	lut_tak
13-Oct-23	17:18:00	20231013_DJI_03	a02	L137	L002	3_7	lut_sim
13-Oct-23	17:38:00	20231013_DJI2_03	a01	L001	L137	3_7	lut_sim
16-Oct-23	15:42:00	20231016_DJI_01	a02	L421	L137	4_5	lut_tak
16-Oct-23	15:59:00	20231016_DJI2_01	a01	L137	L004	4_5	lut_suz
16-Oct-23	15:59:00	20231016_DJI2_01	a02	L137	L575	4_5	lut_tak
16-Oct-23	17:07:00	20231016_DJI2_02	a01	L137	L001	4_5	lut_sim
16-Oct-23	17:07:00	20231016_DJI2_02	a02	L137	L002	4_5	lut_sim
17-Oct-23	14:17:00	20231017_DJI_01	a01	NA	L004	1_4	suz
27-Jun-24	14:51:00	20240627_DJI2_01	a01	NA	L077	2_5	lut
3-Jul-24	14:45:00	20240703_DJI_02	a01	NA	L137	1_4	lut
10-Jul-24	14:45:00	20240710_DJI_02	a01	NA	L137	1_4	lut

SI References

1. A. Bentzur, *et al.*, Early life experience shapes male behavior and social networks in *Drosophila*. *Current Biology* **31**, 486-501.e3 (2021).
